# Oxygen vacancies induced band gap narrowing for efficient visible-light response in carbon-doped TiO_2_

**DOI:** 10.1038/s41598-023-39523-6

**Published:** 2023-08-29

**Authors:** Sujun Guan, Yanling Cheng, Liang Hao, Hiroyuki Yoshida, Chiaki Tarashima, Tianzhuo Zhan, Takaomi Itoi, Tangbin Qiu, Yun Lu

**Affiliations:** 1 Research Center for Space System Innovation, Tokyo University of Science, Chiba, Japan; 2 Beijing Key Laboratory of Biomass Waste Resource Utilization, Beijing Union University, Beijing, China; 3 College of Mechanical Engineering, Tianjin University of Science and Technology, Tianjin, China; 4 Chiba Industrial Technology Research Institute, Chiba, Japan; 5 Bio-Nano Electronics Research Centre, Toyo University, Saitama, Japan; 6 Graduate School and Faculty of Engineering, Chiba University, Chiba, Japan

**Keywords:** Photocatalysis, Computational methods

## Abstract

The band gap of rutile TiO_2_ has been narrowed, via the formation of oxygen vacancies (OVs) during heat treatment in carbon powder (cHT) with embedding TiO_2_ coatings. The narrowed band gap efficiently improves the visible light response of TiO_2_ coatings, to further enhance the visible-light-driven photocatalytic activity. The change in OVs during cHT has been studied by manipulation of cHT temperature and time. The effect of OVs on the band structure of nonstoichiometric TiO_2-*x*_ has been further calculated by first-principles calculations. With raising the temperature, SEM images show that the nano-size fiber-like structure forms on the surface of TiO_2_ coatings, and the amount of the fiber-like structure significantly increases and their size changes from nano to micro under 800 °C, contributing to cause an increase in accessible surface area. The UV–Vis results reveal that the band gap of TiO_2_ has been narrowed during cHT, due to the formed oxygen vacancies. The XPS results further confirm that the formation of surface defects including OVs, and the XPS depth profile further shows the decreased relative amount of O whereas increased relative amount of carbon. Notably, after cHT for TiO_2_ coatings, the photocatalytic activity first increases then decreases with raising the temperature, achieving approximately 3 times at 850 °C. The first-principles calculation suggest that the OVs in TiO_2_ coatings with localized electrons could facilitate the band gap narrowing, further favoring to enhance the photocatalytic activity under visible light.

## Introduction

Face to these urgent issues of environmental pollution and energy crisis, the use of renewable resources to develop renewable energy technologies is becoming an urgent subject that needs breakthroughs. Photocatalysis is considered as a candidate with great potential to alleviate and further solve these problems, because it can efficiently decompose organic pollutants or generate chemical energy via photocatalytic effect. Titanium dioxide (TiO_2_) can be regarded as one of the most important photocatalysts materials, because of the suitable band edge positions, outstanding stability, inexpensive and excellent photocatalytic activity^1–3^. However, TiO_2_ has been limited by its relative wide band gap (~ 3.2 eV of anatase or ~ 3.0 eV of rutile), for the visible light response^4–6^. Currently, tremendous efforts have been dedicated to improving the visible light absorption of TiO_2_, such as narrowing band gap with introduction of dopant or defects (OVs, Ti^3+^, lattice strains), and compositing with photocatalysts having smaller band gap or better visible light response^7–12^, to further meet the needs of practical applications. In addition, many pieces of literatures have reported that doping non-metals such as nitrogen, sulfur, or carbon into TiO_2_ lattice can extend the absorption edge from UV region to visible region, owing to the introduction of localized electronic states in the band gap^13–16^.

Notably, controllable doping of C impurities into TiO_2_ lattice is an effective approach to improve the photocatalytic activity, because that C can permeate into TiO_2_ lattice to substitute O or Ti atoms, accompanied by the formation of Ti–C or C–O–Ti bonds to generate hybrid orbitals above the valence band of TiO_2_, significantly inhibiting the recombination due to the transport channel and electron storage capacity. After Khan et al. reported the carbon substitutes for the lattice oxygen atoms in TiO_2_ with a lower band gap about 2.32 eV in 2002^17^, many researchers have successively proven that C doped TiO_2_ is an effective strategy to narrow the band gap and generate surface defects such as OVs, to further enhance the visible-light-driven photocatalytic activity^18–22^.

Apart from doping, OVs-induced photocatalysis is another efficient solution to achieve visible-light response, charge separation simultaneously, to serve as active site for the adsorption and subsequent dissociation of O-containing molecules. In 2000, Nakamura et al. investigated the role of OVs in TiO_2_ photocatalyst that locate between the valence and the conduction bands, for visible light response^23^, then Schaub et al. unraveled the diffusion mechanism of OVs on rutile TiO_2_ in 2002^24^. Wendt et al. reported that OVs could significantly promote the charge separation, by the induced hole trapping^25^. From the perspective of electronic structure analysis, it is generally believed that OVs can improve the electronic structure of metal oxide semiconductors with introducing some kinds of defect states (such as Ti^3+^, point defect) below the conduction band and narrowing the band gap for better visible-light response^26,27^. More important, the amount of OVs plays a key role on the visible light response and improved photocatalytic activity of TiO_2_ from experimental measurements and theoretical calculations^28–31^.

In this work, to investigation of the formed OVs during cHT and further enhance the photocatalytic activity of TiO_2_, we focus on adjusting the OVs in C-doped TiO_2_ by manipulation of cHT temperature and time. It reveals that the formation of OVs in the surface is significant with raising cHT temperature, compared with that of extending cHT time. In addition, the influence of cHT on the crystal structure, surface morphology, and their photocatalytic activity is analyzed and revealed.

## Experimental

### Fabrication of TiO_2_ and C-doped TiO_2_ coatings

The fabrication of photocatalyst coatings was carried out by coating formation, oxidation and cHT process. Firstly, Ti coatings were formed on Al_2_O_3_ balls (93.0% for purity, 1 mm for average diameter, Nikkato) by machinal coating technique, using Ti powder (99.1% for purity, 30 μm for average diameter, Osaka Titanium technologies), donated as "Ti". The details of MCT operation refer to the previous studies^32–35^. Then, the as-prepared Ti coatings were oxidized at 800 °C for 15 h in the atmosphere with a furnace (HPM-1G, As one), to generate rutile TiO_2_, donated as "TiO_2_". Followed the oxidation, the cHT for TiO_2_ coatings were conducted with embedding in carbon powder (150 μm for average diameter) using a short alumina pipe, and annealed with the same electric furnace, at *x* (650–1000 °C, with an interval of 50 C) for 0.1, 0.5 and 1 h, respectively. The samples were kept in the furnace until approximately 25 °C, and donated as "cHT-*x*℃*y*h", where *x* is the temperature and *y* is the time during the cHT.

### Characterization

The compounds and phase change of the prepared photocatalyst coatings were examined by an X-ray diffraction (XRD, JDX-3530) with Cu-K*α* radiation, with the 2*θ* range from 23 to 65 deg (the step is 0.02 deg/s). The evolutions of surface morphology were observed using a scanning electron microscopy (SEM, Hitachi-SU8030). The chemical composition on the surface were investigated using X-ray photoelectron spectroscopy (XPS, PHI Quantes). UV–Vis absorption spectra were recorded using an ultraviolet–visible spectrophotometer (DRUV-vis, Shimadzu 3700/3700DUV). The evaluation of photocatalytic activity was carried out with the photodecomposition of methylene blue (MB) solution under visible light and UV irradiation at approximately 25 °C. According to the ISO 10678-2010, all samples were firstly dried under UV light for 24 h, then the potential absorption was avoided by soaking within MB solution (20 μmol/L, 35 mL) for 18 h in dark. The samples were evenly laid on the bottom of the cell for the photodecomposition test, and the initial concentration of MB solution was 10 μmol/L. The irradiation intensity of visible light (λ greater than 420 nm) and UV on the samples were set as 5000 lx and 1.0 mW/cm^2^, respectively. The absorbance of MB solution was measured by a colorimeter (mini Photo 10, Sanshin), with the absorption peak around 660 nm. The details of the photocatalytic evaluation can be found in our published works^33–35^. The difference in degradation constant (*R*) between *k*_sample_ and *k*_MB-solution_ was further used to highlight the photocatalytic activity of the samples.

### Computational methods

To further investigate the relationship between surface OVs in TiO_2_ lattice and band gap, the electronic structure calculations were performed to calculate rutile TiO_2-*x*_ surfaces with different contents of OVs by the PHASE based on the framework of density functional theory (DFT)^36,37^, within a program code using plane-wave basis sets based on the pseudopotential method. The difficulty is to alter the stoichiometry of TiO_2-*x*_ that determines the electronic band structures^38^. Consequently, the electronic structure calculations of rutile TiO_2_ and nonstoichiometric TiO_2-*x*_ (TiO_1.958_, TiO_1.917_, TiO_1.875_, and TiO_1.600_) were carried out and investigated. The details of condition calculations refer to the previous studies^37^. The structure parameters of both TiO_2-*x*_ are shown in Supplementary Table S1. A plane-wave cutoff with 340 eV was applied during these calculations, while the convergence in self-consistent field was 27 × 10^–7^ eV. Supplementary Table S2 presents the related main parameters for the calculations.

## Results and discussion

### Crystal structure

**Figure 1:**
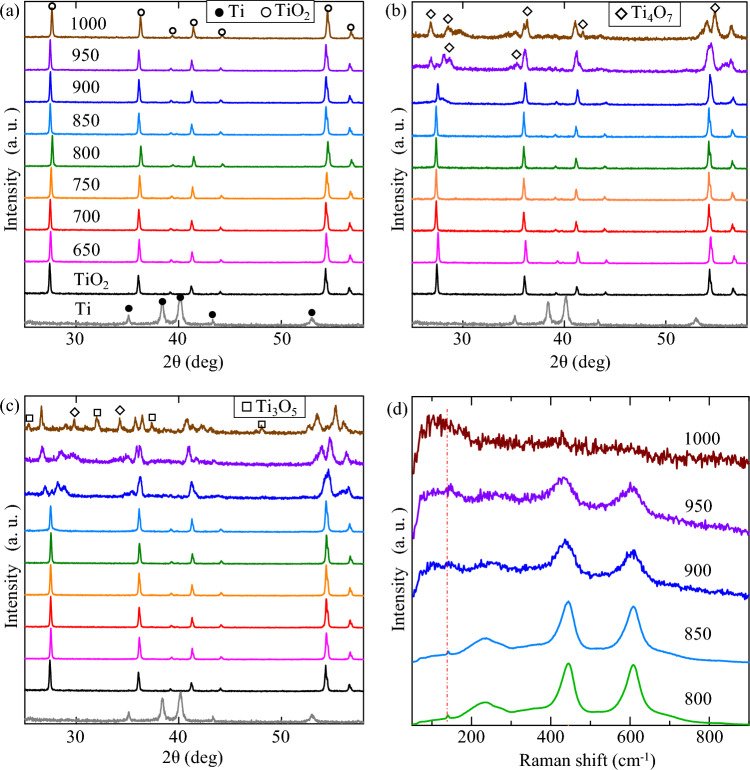
XRD patterns and Raman spectra of the samples. (**a**) cHT-*x*℃0.1 h, (**b**) cHT-*x*℃0.5 h, (**c**) cHT-*x*℃1 h, and (**d**) cHT-*x*℃0.5 h.

XRD patterns of the as-prepared samples are presented in Fig. 1, together with the Raman results of the cHT-*x*℃0.5 h samples. From the Ti sample, it could find that Ti coatings (approximately 50–70 μm^39,40^) were coated on each Al_2_O_3_ ball. While the diffraction peaks at 27.4°, 36.1°, 41.2°, and 54.3° from the TiO_2_ samples show that rutile TiO_2_ successfully form on the surface of Ti coatings after oxidization at 800 °C for 15 h. Figure 1a, with raising the cHT temperature for relative short time of 0.1 h. However, it hard to observe the change even at high temperature of 1000 °C. While extending the cHT time to 0.5 h, the rutile TiO_2_ keeps until the temperature up to 850 °C, then it starts to change, especially at peak of 27.4°, which indicates that the new compounds exhibited Magneli phases that appear to have formed^29,37,41–44^. To further investigate the cHT time at different temperature, the change in crystal structure of the cHT-*x*℃1 h samples is similar to those of the cHT-*x*℃0.5 h samples. The Magneli phases of Ti_4_O_7_ and Ti_3_O_5_ become to replace rutile at 1000 °C for 1 h, as shown in Fig. 1. It could conclude that the influence on rutile TiO_2_ becomes to be found from cHT temperature of 900 °C, simultaneously the cHT time also needs to have a minimum limit. In addition, the grain sizes of the cHT-*x*℃0.5 h samples increase before the formation of Magneli phases (Supplementary Table S3). Similar to that of the XRD result, it is hardly to find the change in Raman spectra from the cHT-*x*℃0.1 h samples (Supplementary Fig. S1), owing to the too short cHT time. It starts to change at 900 °C for at least 0.5 h. Back to the cHT-*x*℃0.5 h samples, Fig. 1d shows that the rutile TiO_2_ significantly starts to change at 900 °C then to be Magneli phases at 1000 °C^45,46^, which is also well matched with those of XRD results.

### Surface morphology evolution

**Figure 2:**
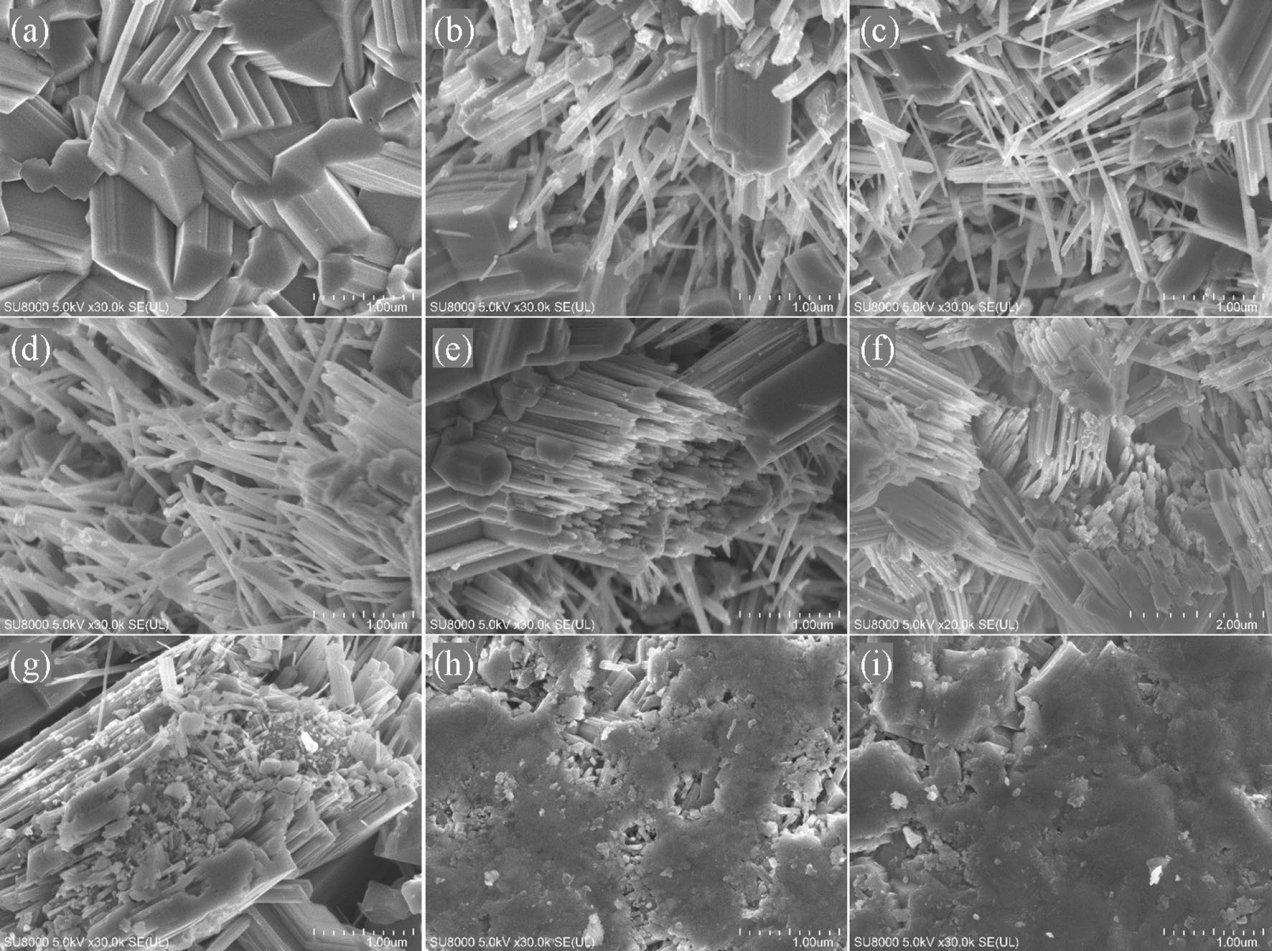
Surface morphology comparison of the TiO_2_ and cHT-*x*℃0.5 h samples. (**a**) TiO_2_, (**b**) cHT-650℃0.5 h, (**c**) cHT-700℃0.5 h, (**d**) cHT-750℃0.5 h, (**e**) cHT-800℃0.5 h, (**f**) cHT-850℃0.5 h, (**g**) cHT-900℃0.5 h, (**h**) cHT-950℃0.5 h, and (**i**) cHT-1000℃0.5 h.

Figure 2 presents the surface morphology evolution of the cHT-*x*℃0.5 h samples. It could be observed that a typical columnar structure of rutile TiO_2_ has been formed, as shown in Fig. 2a ^29,32–35,41,46,47^. Compared to that of TiO_2_, it is clearly found that the change in surface morphology of the cHT-*x*℃0.5 h samples, with raising the cHT temperature. Interesting, a large amount of nano-size needle-like structures are formed during the cHT, with a temperature below 900 °C. From Fig. 2b,c, the needle-like structures are mixed with the columnar structure. With further raising the temperature, the nano-size needle-like structures grow in number and size. Compared with Fig. 2a–g, it reveals that the nano-size needle-like structures are generated with breaking of the columnar structure. While the temperature is higher than 900 °C, due to the generated Magneli phases, a completely different morphology has been formed, replacing those of the needle-like and columnar structures. With the change in the surface morphology caused by the formed compounds and their crystal structures during cHT, it inevitably has a direct impact on its accessible area, which in turn has an impact on the photocatalytic performance.

### UV–Vis absorption and band gap analysis

**Figure 3:**
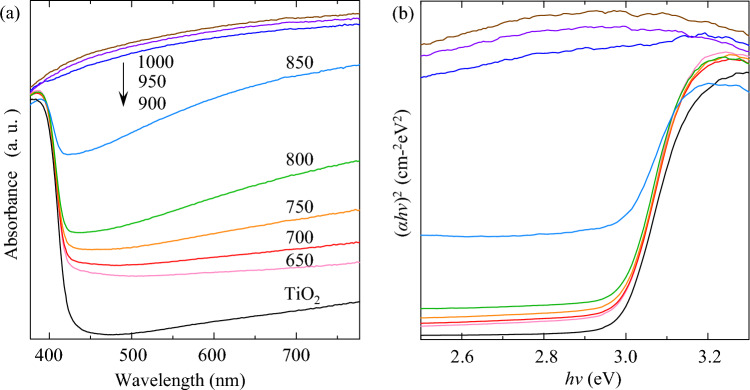
UV–Vis absorption spectra (**a**) and plot of (*αhv*)^2^ against *hv* of the cHT-*x*℃0.5 h samples.

UV–Vis spectroscopy is used to study the electronic states of the cHT-*x*℃0.5 h samples. Figure 3a shows a typical and strong absorbance behavior in UV region and very weak absorption in visible light region, from the TiO_2_ sample. With raising the cHT temperature to 650 and 700 °C, the absorbance edge around 400–420 nm evidently moves towards the visible light range. While further raising the temperature up to 800 °C, the absorbance behavior of the samples strongly increases, especially within the visible region, which could be related to the increased amount of the formed OVs^23–25,28–31^, during cHT with raising the temperature. Notably, in the case of 850 °C, it prominently indicates the significantly moved absorbance edge and strongly increased visible-light response. This proves that cHT is a pivotal role in the absorbance redshift. Moreover, the appearance is more directly show the effect of cHT on the light absorbance (Supplementary Fig. S2). When the temperature is higher than 850 °C, it could find that the absorbance completely changes, attributing to the formed Magneli phases of Ti_4_O_7_ and/or Ti_3_O_5_, having a graphite-like conductivity at room temperature^29,37,42,43^. It means that the band gap of the samples with Magneli phases might be nearly zero eV, which is well matched with the observed absorption from Fig. 3a.

To clearly show the influence of cHT on the band gap, the corresponding Kubelka–Munk plots are shown as Fig. 3b. The change in band gap is slow when the temperature is lower than 800 °C, and the calculated band gap is summarized in Supplementary Table S3. When the temperature is up to 850 °C, the band gap is significantly narrowed to 2.879 eV, from 2.984 of the TiO_2_ sample, due to the generated OVs in lattice of rutile TiO_2_ during cHT.

### Bonding environment

**Figure 4:**
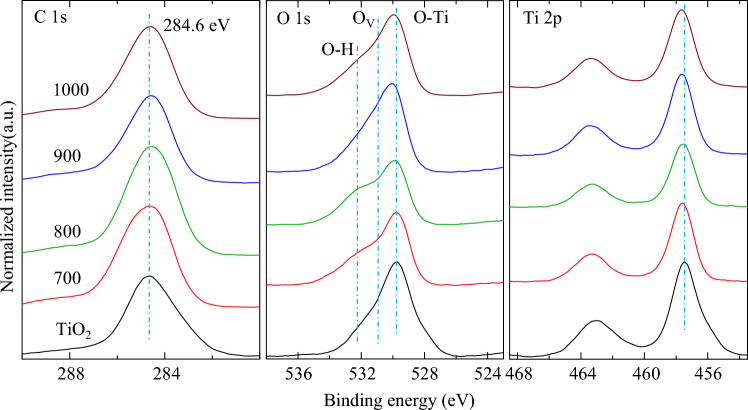
XPS spectra of the cHT-*x*℃0.5 h samples.

XPS spectra was further carried out to study the surface chemical bonding of the cHT-*x*℃0.5 h samples, as illustrated in Fig. 4. In general, the change in C 1 s and Ti 2p is hard to be found, while it is significant in the case of O 1 s, especially in OVs from the cHT-700℃0.5 h and cHT-800℃0.5 h samples. Basically, the peak at approximately 529.8 eV presented with black line in Fig. 4b could be attributed to the O^2−^ ions, usually surrounded by Ti^4+^ ions in rutile TiO_2_^34,47–50^. The asymmetric peaks of O 1 s could be attributable to the three contributions different chemical states, such as the above O^2−^ ions, OVs (around 531.0 eV)^23–25,28–31^ and loosely bound oxygens at grain boundaries or the chemisorbed oxygens (around 532.2 eV)^51–53^. It could be found that a slight change in the O 1 s peak from 529.8 eV of rutile TiO_2_ to 530.0 eV of the cHT-1000℃0.5 h sample, which hints that the possible formation of Ti^3+^ neighboring to OVs in rutile TiO_2_^24–26,29^. The shift in Ti 2p peak also suggests the formation of Ti^3+^^20^. What’s more, the peak change around 531.0 eV incidents that the amount of OVs increase, with raising the cHT temperature. According to the XRD and XPS results, it could conclude that the rutile TiO_2_ on the outer surface starts to react with the involved carbon and its compounds during cHT at 700 °C, while the increased amount of OVs formed and discussed into the bulk of TiO_2_ films at higher temperature, which will affect the crystal structure (Fig. 1).

**Figure 5:**
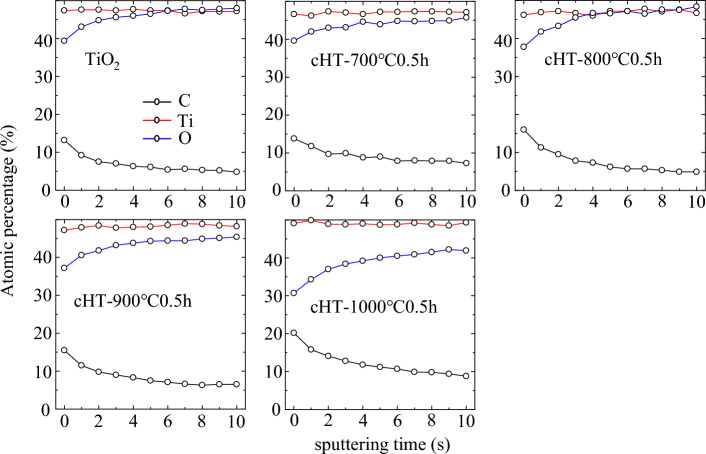
XPS depth profile of the cHT-*x*℃0.5 h samples.

Furthermore, to better understand the effect of cHT for TiO_2_ films on the change in surface elements, XPS depth profile measurements were performed on the cHT-*x*℃0.5 h samples, giving the atomic percentage of namely C, Ti, and O, as shown in Fig. 5. The results indicant that the changes in C and O are significant in the outer surface, whereas Ti is relatively stable. Notably, it shows that the relative amount of C increases and O decreases in the outer surface with raising cHT temperature, revealing the C has successfully doped into the surface of TiO_2_ films and resulted in the formation of OVs easily occurs at higher 800 °C^15–21,34^. While the amount of O from the cHT-1000℃0.5 h sample drops below 40% and is significantly lower compared to other samples, which could be attributed to the formation of the Magneli phase.

### Photocatalytic activity

**Figure 6:**
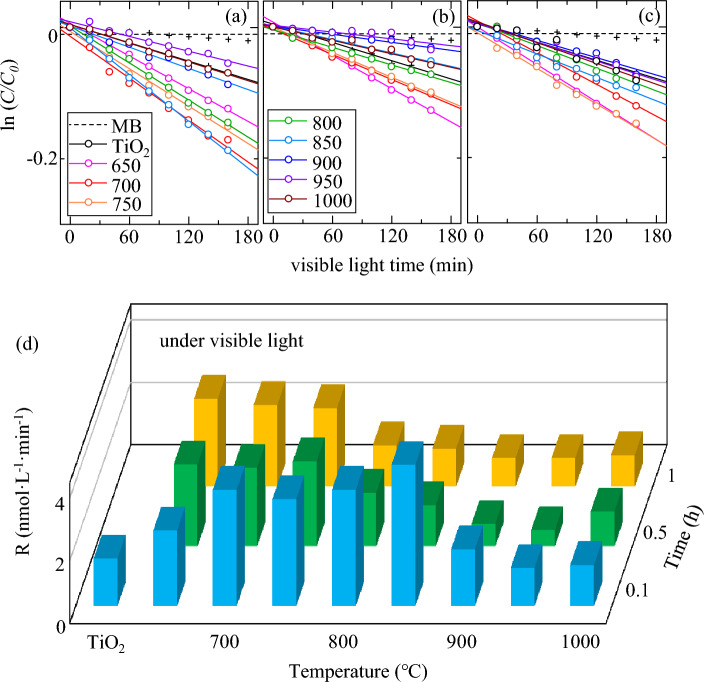
Concentration evolution of MB solution under visible light for the cHT-*x*℃*y*h samples. (**a**) 0.1 h, (**b**) 0.5 h, (**c**) 1 h and (**d**) Degradation constant R between *k*_sample_ and *k*_MB-solution_.

For the photodegradation of MB solution presented with the samples, Fig. 6 clearly reveals that the cHT could efficiently enhance the visible-light-driven photocatalytic activity (Supplementary Fig. S3), and same in the case of UV irradiation (Supplementary Fig. S4). The hardly decreased blank dotted line hints that the MB solution is relatively stable. In general, with raising the cHT temperature or extending the cHT time, it demonstrates that the photocatalytic activity firstly increases then decreases due to the concentration change of MB solution. Compared with that of extending the cHT time, it seems that raising the cHT temperature is more efficiently improve the photocatalytic activity with 0.1 h. Figure 6d more directly shows that the changes in degradation constant (R), with all cHT condition. In the case of 0.1 h, the photocatalytic activity keeps enhancing until the cHT temperature reached 850 °C, whereas for the longer cHT time of 0.5 h and 1 h, the photocatalytic activity starts to decrease when the temperature is higher than 800 °C. In other words, the photocatalytic activity could be enhanced at a relative higher temperature for shorter time. It is well known that the doping of metal or nonmetal ions is often accompanied by formation of OVs in the lattice of TiO_2_^18–21^. Numerous studies have reported that the presence of OVs in TiO_2_ can located at 0.75–1.18 eV below the conduction band minimum, to effectively expand the visible light absorption range^7–9,23–25^. The enhanced visible-light-driven photocatalytic activity could be attributed to the visible-light response due to the narrowed band gap (especially under 850 °C in Fig. 3), and the increased accessible area due to the formed nano-size needle-like structures on the surface until disappearing around 850 °C (Fig. 2). Correspondingly, when the temperature is higher than 850 °C, the decreases in the photocatalytic activity at are mainly because of the formed Magneli phases (Fig. 1)^29,54,55^, and the destroyed needle-like structures on the surface.

While comparing the cHT time under different temperature, the time is necessary at lower temperature of 650 °C, then the temperature will dominant the enhancement of visible-light-driven photocatalytic activity, compared with that of time. Notably, the advantage of cHT temperature for 0.1 h is more prominent above 800 °C, and the R of cHT-850℃0.1 h is further improves by about 3 times, compared with that of TiO_2_. Moreover, the photocatalytic activity under UV irradiation could be further enhanced at 800 to 850 °C for 0.5 h.

### Simulation ab

**Figure 7:**
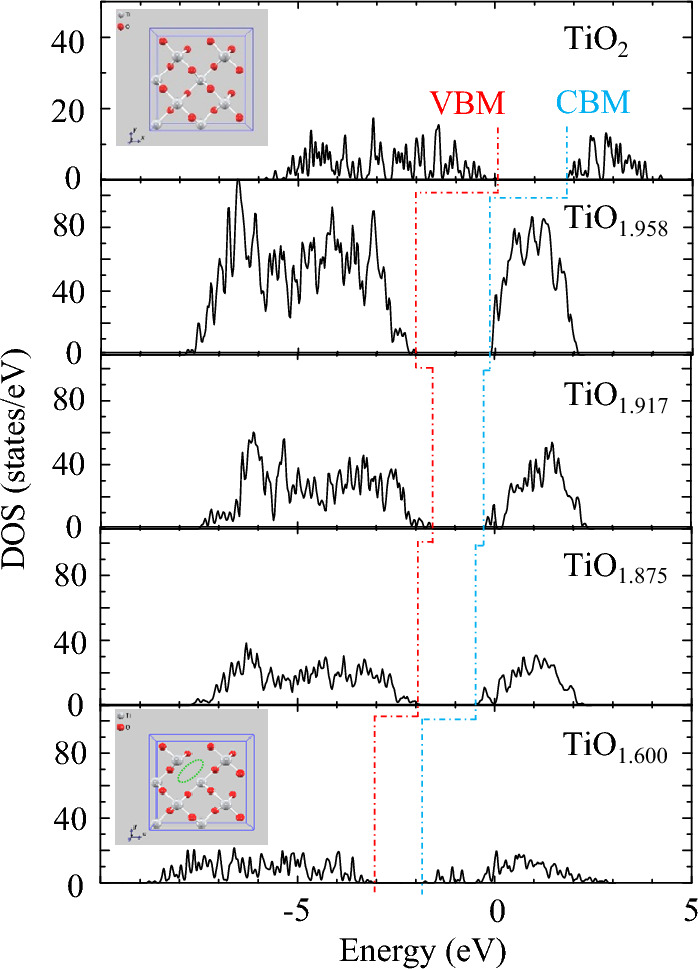
TDOS of TiO_2_ and TiO_2-*x*_ near Fermi level. The Fermi level during the calculation was set as zero. The red dotted line represents valence band maximum (VBM), while the blue dotted line is conduction band maximum (CBM).

To further investigate the effect of OVs on the visible-light response of TiO_2_, the DFT method was adopted to calculate the stoichiometric and non-stoichiometric rutile TiO_2_ (110) surfaces. The effect of OVs on the total density of states (TDOS) of TiO_2-*x*_ (*x*: 0, 0.042, 0.083, 0.125 and 0.400) are presented in Fig. 7. In general, a high vacancy concentration could induce a vacancy band of electronic states just below the conduction band, which had been confirmed by theoretical calculations and relevant experiments^37,56–58^. Interestingly, Fermi level significantly shifts toward the conduction band with the increased amount of OVs, which indicates that the introduction of OVs results in two loosely captured electrons by three Ti dangling bonds^23,27,29,37,56^. Moreover, the band gaps of TiO_2-*x*_ start to decrease from TiO_1.917_, by about 0.02–0.5 eV with respect to 1.88 eV from pure TiO_2_, which is consistent with previous study^37,57,58^.

## Conclusions

In this study, the OVs induced visible-light response C-doped TiO_2_ photocatalyst films with were successfully prepared by a simple and efficient method of cHT for TiO_2_ formed on Ti coatings. The visible-light-driven photocatalytic activity of C-doped TiO_2_ has been significantly enhanced by around 3 times, compared with that of pure TiO_2_. With raising the cHT temperature under 850 °C, the surface morphology with nano-size needle-like structures forms that could significantly increase the accessible surface area, while the needle-like structures would disappear at higher temperature. Generally, with raising the cHT temperature or extending the cHT time, it demonstrates that the photocatalytic activity firstly increases then decreases, reaching a better photocatalytic activity under 850 °C for 0.1 h. The enhanced visible-light-driven photocatalytic activity could be attributed to the improved visible-light response due to the narrowed band gap, and the increased accessible area due to the formed nano-size needle-like structures. Compared with cHT time, the photocatalytic activity could be enhanced at a relative higher temperature for shorter time. Notably, the introduction of OVs results in substantially narrowing band gap of TiO_*2-x*_, compared with that of pure TiO_2_.

### Supplementary Information

Supplementary Information 1.

Supplementary Information 2.

## Data Availability

All data generated or analysed during this study are included in this published article (and its supplementary information files).
