# Supplementary material for: Oxygen vacancies induced band gap narrowing for efficient visible-light response in carbon-doped TiO2
Source: Sci Rep. 2023 Aug 29;13:14105. doi: 10.1038/s41598-023-39523-6 (PMC10465500; doi:10.1038/s41598-023-39523-6)
Supplement: Supplementary file 2 — Supplementary Information 2. [file 41598_2023_39523_MOESM2_ESM.doc]

**Oxygen vacancies induced narrow bandgap for efficient visible-light response in carbon-doped TiO_2_**

Sujun Guan^a^, Yanling Cheng^b,*^, Liang Hao^c^, Hiroyuki Yoshida^d^, Chiaki Tarashima^a^, Tianzhuo Zhan^e^,

Takaomi Itoi^f^, Tangbin Qiu^f^, Yun Lu^f,*^

**Table S1.** Structure parameter of TiO_2_ and TiO_2-_*_x_* in Bravais lattice.

| **Formula** | **a axis (nm)** | **b axis (nm)** | **c axis (nm)** | **Num. Ti** | **Num. O** | **Num. unit cell** |
| --- | --- | --- | --- | --- | --- | --- |
| TiO_2_ | 0.4593 | 0.4593 | 0.2959 | 2 | 4 | 1 |
| TiO_1.958_ | 0.9186 | 0.9186 | 0.8877 | 24 | 47 | 12 |
| TiO_1.917_ | 1.3779 | 0.9186 | 0.2959 | 12 | 23 | 6 |
| TiO_1.875_ | 0.9186 | 0.9186 | 0.2959 | 8 | 15 | 4 |
| TiO_1.600_ | 0.9186 | 0.4593 | 0.2959 | 5 | 8 | 2 |
| Ti_4_O_7_ | 0.5597 | 0.6903 | 0.7125 | 8 | 14 | 2 |

**Table S2.** Condition parameter of the first principal calculation.

| **Formula** | **Cut-off** | | | **K point sampling mesh** | **Solver** | **Convergence (eV)** |
| --- | --- | --- | --- | --- | --- | --- |
|  | **Wave function (eV)** | **Charge density (eV)** | **Num. bands** |  |  |  |
| TiO_2_ | 520 | 4680 | 29 | 3×3×5 | Mdd+rmm3 | 2.7E-5 |
| TiO_1.958_ | 520 | 4680 | 343 | 2×2×2 | Mdd+rmm3 | 2.7E-4 |
| TiO_1.917_ | 520 | 4680 | 170 | 2×2×3 | Mdd+rmm3 | 2.7E-4 |
| TiO_1.875_ | 520 | 4680 | 112 | 3×3×5 | Mdd+rmm3 | 2.7E-4 |
| TiO_1.600_ | 520 | 4680 | 65 | 2×3×5 | Mdd+rmm3 | 2.7E-5 |
| Ti_4_O_7_ | 520 | 4680 | 109 | 3×2×2 | Mdd+rmm3 | 2.7E-4 |

**Fig. S1** Raman spectra of the samples.

(a) cHT-*x*℃0.1h, (b) cHT-*x*℃0.5h, and (c) cHT-*x*℃1h.

**Table S3.** Comparison of band gap, grain size and oxygen ratio of the cHT-*x*℃0.5h sample.

| **Sample** | **Band gap (eV)** | **Grain size (nm)** | **O-Ti (%)** | **OV (%)** | **O-H (%)** |
| --- | --- | --- | --- | --- | --- |
| TiO_2_ | 2.984 | 72.2 | 83.46 | 0.00 | 16.54 |
| R-650℃0.5h | 2.979 | 74.6 |  |  |  |
| R-700℃0.5h | 2.977 | 70.7 | 80.14 | 0.98 | 18.88 |
| R-750℃0.5h | 2.973 | 72.7 |  |  |  |
| R-800℃0.5h | 2.968 | 77.9 | 78.00 | 4.65 | 17.35 |
| R-850℃0.5h | 2.879 | 77.6 |  |  |  |
| R-900℃0.5h | - | 74.5 | 56.98 | 24.53 | 18.49 |
| R-950℃0.5h | - | 36.2 |  |  |  |
| R-1000℃0.5h | - | 28.0 | 65.16 | 12.46 | 22.38 |

**Fig. S2** The appearance of the cHT-*x*℃*y*h samples.

**Fig. S3** Photocatalytic activity of the cHT-*x*℃*y*h samples towards MB solution under visible light. (a,) 0.1, (b) 0.5, (c) 1.

**Fig. S4** Photocatalytic activity of the cHT-*x*℃*y*h samples towards MB solution under UV light. (a, b) 0.1, (c, d) 0.5, (e, f) 1 and (g) Degradation constant.
